# Range of Motion and Muscle Strength Changes in Japanese Professional Pitchers During the Baseball Season

**DOI:** 10.7759/cureus.49844

**Published:** 2023-12-02

**Authors:** Yasuhiko Sumimoto, Shin Yokoya, Akihiko Kitasaka, Yohei Harada, Masataka Deie, Nobuo Adachi

**Affiliations:** 1 Orthopaedic Surgery, Graduate School of Biomedical and Health Sciences, Hiroshima University, Hiroshima, JPN; 2 Orthopaedic Surgery, Hiroshima City Hiroshima Citizens Hospital, Hiroshima, JPN; 3 Physical Therapy, Mazda Hospital, Hiroshima, JPN

**Keywords:** season progress, isometric muscle strength, motion range, professional baseball players, throwing disorder

## Abstract

Background: There are no studies examining changes in the physical function throughout the baseball season in Japanese professional players. We examined the range of motion (ROM) and muscle strength changes in professional baseball pitchers as the season progresses.

Materials and methods: Five professional baseball pitchers were included. ROM, flexibility, and muscle strength of the trunk and shoulder, elbow, hip, knee, and ankle joints were measured pre-season (PRE), mid-season (MID), and post-season (POST).

Results: The total shoulder arc ROM of the dominant sides significantly decreased compared to that of the nondominant sides at MID and POST. Shoulder abduction muscle strength of the dominant sides significantly decreased at POST compared to that at PRE. In the trunk, lateral flexion ROM of bilateral sides significantly decreased at MID and POST compared to that at PRE, and the duration time of the side bridge test of the dominant sides significantly decreased at POST to that at PRE. Hip extension ROM and muscle strength and internal rotation ROM of the push-off leg significantly decreased at POST compared to that at PRE.

Conclusion: The total shoulder arc ROM and shoulder abductor muscle strength of the dominant sides, trunk lateral flexion ROM and muscle strength, push-off leg hip joint extension muscle strength and ROM, and internal rotation ROM were more susceptible to changes as the season progressed. In order to maintain performance and prevent a throwing disorder, it is necessary to focus on these movements during the season and to appropriately train and condition these muscle groups.

## Introduction

Baseball is one of the most commonly played sports. The throwing movement is particularly complicated among baseball players, and poor throwing movements increases the risk of throwing disorder [1]. The throwing motion is very fast, with the internal rotation angle velocity of the shoulder joint reaching up to >8000 degrees/second and the extension angle velocity of the elbow joint reaching up to >2500 degrees/second [2]. Moreover, the maximum external rotation angle at the shoulder joint, including the scapula and thorax, at the late cocking phase exceeds 180°; the stress applied to the shoulder joint reaches >1000 N and >900 N at the elbow joint [2,3]. Therefore, the throwing motion seems to over-stress the shoulders and elbows of baseball players. Since professional baseball pitchers, in particular, have long seasons, consisting of more than 140 games over a season of seven months, the fatigue may accumulate in the latter half of the season.

In a previous study, there was no change in the muscle strength of the shoulder internal and external rotators in university baseball pitchers before and after the season [4]; however, there are no studies examining the changes of the range of movement (ROM) and muscle strength throughout the season in professional baseball pitchers.

We hypothesized that ROM and muscle weakness of not only the shoulder joint but the whole body would decrease as the season progresses. Thus, we aimed to examine the ROM and muscle strength changes in Japanese professional baseball pitchers as the season progressed.

## Materials and methods

This study was a case series, approved by the Ethical Committee for Epidemiology of Hiroshima University (approval no. E-1206), and conducted in accordance with the principle of the Declaration of Helsinki. Written informed consent was obtained from the participants. Pitchers who belonged to one professional baseball team, provided consent for the study, and were available for medical checkups throughout the season were included. Players who experienced shoulder pain or disability during the season were excluded. Finally, five professional baseball pitchers who had no restrictions on practice or competing at the time of measurement were included in the study. Table 1 shows the basic information of the participants. This study included farm pitchers, two starting and three set-upper pitchers, and the throwing types were four overhand-throwing and one side-throwing.

**Table 1:** Basic information of the participants SD: standard deviation

Characteristics	Mean ± SD, n
Age (y)	25.0 ± 2.3
Sex (male / female)	5 / 0
Height (cm)	185.0 ± 4.3
Weight (kg)	84.6 ± 7.9
Dominant side (right / left)	3 / 2

The measurement periods were pre-season (PRE), mid-season (MID), and post-season (POST). PRE was defined as March-April, MID as July-August, and POST as November. All the pitchers threw approximately 10 pitches the day before the measurement and did not throw on the day of the measurement. All measurements were conducted in the morning at Hiroshima University Hospital, Hiroshima, Japan.

ROM measurement

We evaluated the ROM and flexibility of bilateral joints (shoulder, elbow, trunk, hip, knee, and ankle) each time. Measurements were taken by two examiners simultaneously, one moving the joint and the other measuring the angle and distance. The order of measurements was always the same for all pitchers.

In the shoulder joints, posterior shoulder tightness was assessed according to previous reports [5]. We measured the abduction angle (AA) and horizontal flexion angle (HFA), while the examiner fixed the lateral border of the scapula [5]. The more restricted ROM on the dominant side compared to the nondominant side, the greater the posterior shoulder tightness is considered. The ROM of the external and internal rotations in 90° shoulder abduction were measured to the end range with the scapula stabilized. In determining the end range of motion of internal rotation, measurements were taken where the examiner's fingers felt pressure on the coracoid process during the rotation [6]. The total arc ROM was calculated using the recorded external and internal rotations. The flexion and extension ROM at the elbow joint were measured. All measurements were performed in the supine position.

The rotation and lateral flexion ROMs of the trunk were measured bilaterally in the sitting position. Both were measured with arms crossed in front of the chest.

In the lower limb, flexion, extension, abduction, adduction, and external and internal rotation ROM of the hip and straight leg raising were measured. The heel-buttock distance as thigh flexibility and the plantar- and dorsiflexion ROMs of the ankle joint were measured. The pelvis was manually fixed to prevent compensatory movements; the hip rotations and heel-buttock distance were measured in the prone position and the others in the supine position.

Muscle strength measurement

Maximum isometric muscle strengths were measured using a handheld dynamometer (microFET2, Nihon Medix Co., Ltd., Chiba, Japan). All measurements were performed twice by the same examiner, and the average value was used.

In the upper limb, external and internal rotations at 90° abduction, shoulder abduction, and movements of the middle and lower fibers of the trapezius were measured at the shoulder joint. External and internal rotations were measured with the shoulder in 90° abduction, elbow joint in 90° flexion, and forearm in the mid-supine position. Shoulder abduction-muscle strength was measured with the shoulder abducted to 90°, horizontally flexed by 45°, and with the forearm in mid-prone while in the seated position [7]. The trapezius middle fiber strength was measured with the shoulder abducted to 90° and maximum forearm supination while in the prone position; the trapezius lower fiber strength was measured with the shoulder abducted to 145° and maximum forearm supination while in the prone position [8,9]. The extension strength of the elbow joint was measured in the prone position.

The trunk muscle strength was measured using a flexor endurance test to hold the trunk in a sitting position with the hip and knee flexed at 90° and the trunk tilted 60° from the floor, a side bridge test in which the pelvis is lifted off a surface and held while keeping the body and elbow straight in the lateral position, and an extensor endurance test to hold the trunk in a horizontal position with the trunk hanging off the bed in the prone position [10].

In the lower limb, the muscle strength of the hip and knee joints were measured based on a previous study [11]. In the hip joint, the muscle strength of the flexors, extensors, abductors, adductors, and rotators were measured. Flexion muscle strength was measured in the end sitting position on the bed, and abduction and adduction were measured while flexing the contralateral lower limb and grasping the bed with bilateral hands in the supine position. Extension and external and internal rotation muscle strengths were measured in the prone position. The extension muscle strength was measured with the knee extended. The external and internal rotation muscle strengths were measured with the knee flexed to 90°. The flexion and extension strengths at the knee joint were measured. The flexion muscle strength was measured with the knee flexed to 90° in the prone position; the extension muscle strength was measured with the knee flexed to 90° using a fixation belt in the end sitting position on the bed. We calculated the hamstrings-to-quadriceps ratio (H/Q ratio), which is the ratio of the knee flexion and extension muscle strength.

Statistical analysis

IBM SPSS Statistics for Windows, version 19 (released 2010; IBM Corp., Armonk, New York, United States) was used for the statistical analysis. Two-way ANOVA was performed using two factors, measured limb and measurement time. The simple main effect test was performed on items for which interaction was observed, and the main effect test was performed on items for which no interaction was observed. In the main effect test, a paired t-test was performed for those with a main effect on the measured limb, and Dunnett’s multiple-comparison method was performed for those with a main effect on the measurement time. Differences were considered statistically significant at p < 0.05. Numerical values are expressed as mean value ± standard deviation. Power analysis was not possible due to the small sample size.

## Results

Season results

The season results are shown in Table 2. During the season, no player left for a long time due to poor conditioning, such as a disorder. The number of games during the season was 27.0 ± 12.7 games and the number of balls was 1206 ± 746 pitches.

**Table 2:** Season results Numerical values are presented as mean value ± standard deviation. The mean number of games, innings, and balls from the start of the season to July-August were measured as MID. The mean number of games, innings, and balls from the start of the season to November were measured as POST. PRE: pre-season, MID: mid-season, POST: post-season

	PRE	MID	POST
Games	-	19.2 ± 8.90	27.0 ± 12.7
Innings	-	53.5 ± 32.0	77.5 ± 47.4
Pitches	-	820 ± 482	1206 ± 746

Changes in the upper-limb ROM

**Figure 1:**
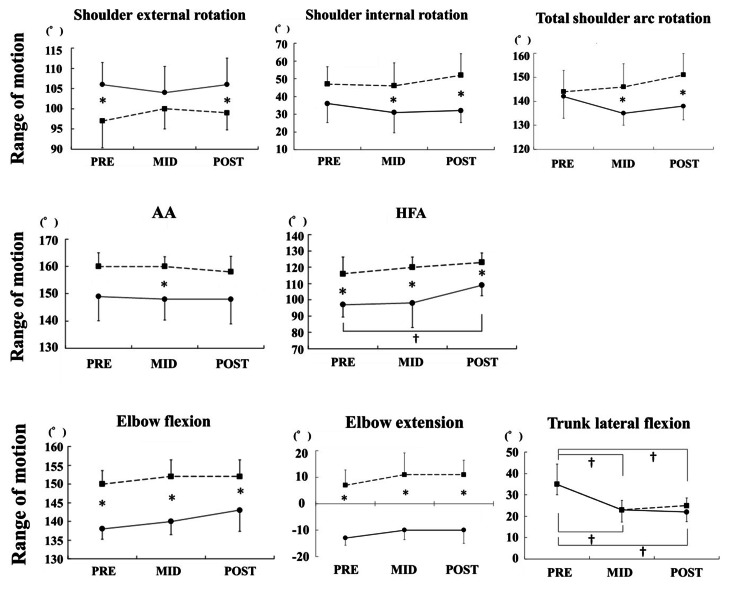
Changes in the range of motion The total shoulder arc ROM on the dominant sides significantly decreased compared to that on the nondominant sides at MID and POST. In the trunk, lateral flexion ROM of bilateral sides significantly decreased at MID and POST compared to at PRE. Hip extension and internal rotation ROM of the push-off leg significantly decreased at POST compared to that at PRE. AA: abduction angle, HFA: horizontal flexion angle, PRE: pre-season, MID: mid-season, POST: post-season. ●: Dominant side, ■: Nondominant side. *, †: P < 0.05

**Figure 2:**
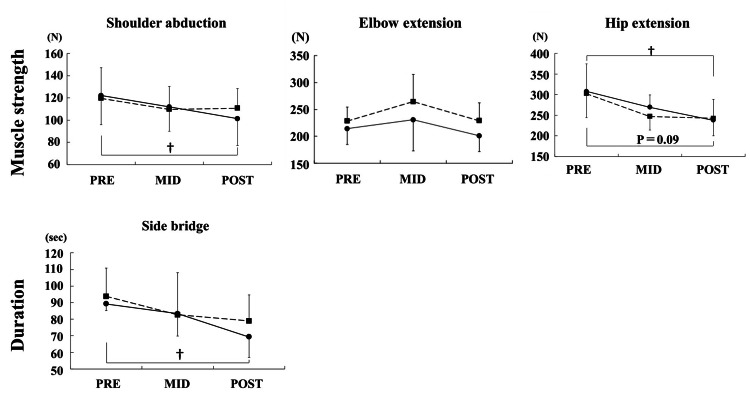
Changes in muscle strength The shoulder abduction muscle strength of the dominant sides significantly decreased at POST compared to that at PRE. The duration of the side bridge test of the dominant sides significantly decreased at POST compared to that at PRE. The hip extension muscle strength of the push-off leg significantly decreased at POST compared to that at PRE. PRE: pre-season, MID: mid-season, POST: post-season. ●: dominant side, ■: nondominant side. †: P < 0.05

Changes in the ROM and muscle strength are shown in Figure 1 and Figure 2, respectively. In the shoulder-joint ROM, there were significant differences in the external rotation at PRE and POST on the affected sides (dominant vs. nondominant side: 106° ± 5.5° vs. 97° ± 6.7° at PRE and 106° ± 6.5° vs. 99° ± 4.2° at POST, p = 0.03 and 0.04, respectively). The internal rotation showed significant differences on the affected sides at MID and POST (dominant vs. nondominant side: 31° ± 11.4° vs. 46° ± 12.9° at MID and 32° ± 6.7° vs. 52° ± 12.0° at POST, p = 0.04 and 0.02, respectively). In the total arc, there were significant differences on the affected sides at MID and POST (dominant vs. nondominant side: 135 ± 5.0° vs. 146 ± 9.6° at MID and 138 ± 5.7° vs. 151 ± 8.9° at POST, p = 0.04 and 0.01, respectively). AA showed significant differences on the affected sides at MID (dominant vs. nondominant side: 148 ± 7.6° vs. 160 ±3.5°, p = 0.01). The ROM of the dominant sides at PRE and POST decreased, although the differences were not significant. For the HFA, there were significant differences on the affected sides at any time (dominant vs. nondominant side: 97 ± 7.6° vs. 116 ± 10.2° at PRE, 98 ± 14.8° vs. 120 ± 6.1° at MID, and 109 ± 6.5° vs. 123 ± 5.7° at POST, p = 0.02, 0.01, and 0.01, respectively), and the dominant sides significantly increased at POST compared to at PRE. In addition, there were significant differences on the affected sides in the elbow flexion and extension at any time (flexion of the dominant vs. nondominant side: 138° ± 2.7° vs. 150 ± 3.5° at PRE, 140° ± 3.5° vs. 152° ± 4.5° at MID, and 143° ± 5.7° vs. 152° ± 4.5° at POST, p = 0.01, 0.01, and 0.03, respectively; extension of the dominant vs. nondominant side: -13° ± 2.7° vs. 7° ± 5.7° at PRE, -10° ± 3.5° vs. 11° ± 8.2° at MID, and -10° ± 5.0° vs. 11° ± 5.5° at POST, p = 0.01, 0.01, and 0.01, respectively). There were no significant differences in the other parameters.

Changes in the trunk and lower limb ROM

In the trunk ROM, the lateral flexion of bilateral sides significantly decreased at MID and POST compared to that at PRE (dominant side at PRE vs. MID and POST: 35 ± 5.0° vs. 23 ± 5.7° and 22 ± 4.5°, p = 0.01 and 0.01, respectively; nondominant side at PRE vs. MID and POST: 35 ± 9.4° vs. 23 ± 4.5° and 25 ± 3.5°, p = 0.03 and 0.02, respectively). In the hip extension and internal rotation, the ROM of the push-off leg significantly decreased at POST compared to that at PRE (extension at PRE vs. POST: 18 ± 2.7° vs. 14 ± 4.2°, p = 0.04; internal rotation: 33 ± 10.4° vs. 25 ± 10.6°, p = 0.01). There were no significant differences in the other items.

Changes in upper limb muscle strength

In the shoulder muscle strength, shoulder abduction of the dominant sides significantly decreased at POST compared to that at PRE (PRE vs. POST: 122.1 ± 25.0 N vs. 101.4 ± 24.2 N, p = 0.03). Although there were no significant differences in the affected sides in the elbow extension, the dominant sides decreased at any time compared with the nondominant sides. There were no significant differences in the other items.

Changes in the trunk and lower limb muscle strength

In the trunk muscle strength, the duration of the side bridge test of the dominant sides significantly decreased at POST compared to at PRE (PRE vs. POST: 89.2 ± 4.0 seconds vs. 69.2 ± 12.4 seconds, p = 0.02). For the lower limb muscle strength, the hip extension muscle strength of the push-off leg significantly decreased at POST compared to that at PRE (PRE vs. POST: 308.0 ± 66.8 N vs. 238.2 ± 37.5 N, p = 0.04), and a similar tendency was also observed in the stride legs (PRE vs. POST: 302.4 ± 58.3 N vs. 242.5 ± 46.3 N, p = 0.09). For the knee joint, there were no significant differences in the measured limbs or times, but the H/Q ratio clinically decreased on bilateral sides (Table 3). There were no significant differences in other items.

**Table 3:** Muscle strength of flexion and extension at the knee joint and H/Q ratio Numerical values are presented as mean ± standard deviation. PRE: pre-season, MID: mid-season, POST: post-season, H/Q ratio: hamstrings-to-quadriceps ratio

	PRE	MID	POST
Flexion (N)			
Dominant side	226.5 ± 31.1	234.1 ± 57.0	176.1 ± 53.9
Nondominant side	219.8 ± 44.8	223.7 ± 43.9	209.5 ± 30.2
Extension (N)			
Dominant side	613.0 ± 80.6	627.8 ± 103	612.5 ± 168
Nondominant side	641.3 ± 105	632.1 ± 97.6	563.3 ± 164
H / Q ratio			
Dominant side	0.37 ± 0.1	0.37 ± 0.1	0.29 ± 0.1
Nondominant side	0.34 ± 0.1	0.35 ± 0.1	0.37 ± 0.1

## Discussion

This study is the first to examine the influence of the baseball season on the progression of physical function of professional pitchers. Although a throwing disorder mainly occurs in the shoulder or elbow joints, it is considered to be a combination of problem at the lesion site and dysfunction of the trunk and lower limb joints. Therefore, it is important to examine which part of the body's ROM and muscle strength are likely to decrease as the season progresses and to know the parts that should be conditioned intensively to maintain performance and prevent occurrence of a throwing disorder.

The shoulder external rotation increased and the shoulder internal rotation and elbow ROM decreased on the dominant side. These results are comparable to previous studies [6,8,9,12]. These are reportedly caused by body changes, such as an increase in the humeral anteversion angle, and soft tissue changes, due to the tightness of joint capsules and muscles [6,12,13], which may have occurred in the dominant upper limbs of this study. Although no significant changes were observed in these ROMs as the season progressed, the difference between the dominant and nondominant sides in the shoulder internal rotation was 20° on average at POST (dominant sides: 32.0 ± 6.7°, nondominant sides: 52.0 ± 12.0°). The limitation of the shoulder internal rotation ROM of 20° increases the risk of internal impingement and superior labral anterior posterior (SLAP) lesion [6,14,15]. AA at MID and HFA at any time of the dominant sides decreased compared to the nondominant sides, and tightness of the lower shoulder joint and posterior tissue was observed. HFA, which is an index of posterior shoulder tightness, is reportedly limited in cases with pathological internal impingement during throwing [15,16]. However, HFA of the dominant sides improved at POST compared to that at PRE in this study. Although HFA changes as the season progress have not been examined, the changes are probably caused by stretching of the horizontal flexors, which are relatively easy compared to the rotators. However, the differences between the dominant and nondominant sides remain (dominant sides: 109° ± 6.5°, nondominant sides: 123° ± 5.7°), and athletes and trainers need to constantly improve the posterior tightness and internal rotation of the shoulder. Moreover, there was no differences between the dominant and nondominant sides at PRE in the total shoulder arc ROM; however, the ROM of the dominant sides was significantly limited at MID and POST compared to that of the nondominant sides. In a previous study of amateur baseball players, there was no difference in the total arc shoulder ROM between the dominant and nondominant sides [8,12]. By contrast, it has been reported that the throwing disorder rate will increase if the total arc shoulder ROM of the dominant sides is limited [6,12]. Therefore, there is a higher risk of developing a throwing disorder in the latter half of the season.

The strength of elbow extension of the dominant sides decreased compared to the nondominant sides each time, although not significantly. The throwing motion, from the maximum shoulder external rotation to the ball release, is mainly performed by elbow extension [2]. Although the muscle strength of the elbow extension is reportedly related to throwing speed [17], its relation to the throwing disorder has not been reported. Weakness of the elbow extension could lead to decrease in the performance. In addition, shoulder abduction muscle strength of the dominant sides significantly decreased at POST compared to that at PRE. Supraspinatus muscle weakness is associated with various shoulder joint disorders, such as rotator cuff injury, impingement syndrome, and SLAP injury [7]. Taking these into consideration, throwing disorders could likely develop in the upper limbs of professional baseball pitchers as the season progresses.

The lateral flexion ROM of the trunk on both sides and the duration of side bridge test on the dominant side decreased as the season progressed. The core muscles provide the core stability to the trunk, and the core stability establishes the basis for the muscle groups that move the limbs [18]. The muscle stabilization mechanism of the trunk is divided into intrinsic and extrinsic muscles. The intrinsic muscles mainly contain short segmental muscles that attach to the spinal column and are involved in the fine coordination of the spinal multi-segments [18]. Therefore, we hypothesized that the intrinsic muscles, such as the intertransversarii muscles, became fatigued early in the season due to the complicated, repetitive, heavy throwing motions, which manifested as a decrease in the trunk lateral flexion ROM. However, since the fatigue could not be quantified in this study, it is still a matter of speculation. There were no significant differences in the trunk rotation ROM in our study. Due to the spinal column structure, the trunk rotation mainly occurs at the thoracic spine, and lateral flexion occurs at the thoracic lumbar spines at almost the same ratio [18]. Hence, we concluded that the limitation of the lateral flexion ROM occurred mainly in the lumbar spine and the trunk rotation ROM did not change. Considering that the trunk bends toward the nondominant side during ball release [19], it forces an eccentric contraction of the dominant side trunk muscles. Eccentric contraction causes greater muscle fatigue and slower recovery than concentric contraction [20]. Therefore, we concluded that the duration of the side bridge test on the dominant sides decreased at POST because the repeated eccentric contractions had affected the intrinsic and extrinsic muscles of the trunk.

It has been reported that the movement of the trunk and pelvis correlates with that of the shoulder joint during the throwing motion [21]. Hence, the decrease in stability due to trunk muscle abnormalities may disrupt the motor chain and increase the burden on the upper limb joints to compensate for it. Although the role of the trunk is considered very important during throwing motions, only the trunk movements during throwing have been studied [21]; there are no studies that have examined the factors limiting trunk function. Therefore, it is necessary to examine the factors affecting trunk function and throwing disorder based on the movements in the future.

In the lower limb, the extension and internal rotation ROM of the push-off leg hip joint decreased in our study. In the push-off leg hip joint, external rotation and extension occur during the cocking period of throwing, and adduction and internal rotation occur during the acceleration period [22]. The scapular and thoracic movements make major contributions to the maximal shoulder external rotation [23], and translation of the pelvis by extending the hip joint is necessary in order to make these prominent. When the hip extension of the push-off leg is limited, the forward translational of the pelvis is prevented, and subsequently, the trunk tilts forward early in the throwing motion. As a result, horizontal extension stress is applied to the glenohumeral joint. The throwing motion requires sufficient hip internal rotation, and insufficient amounts of hip rotation can alter the kinetic chain [24]. These facts suggest that it is important to adjust the condition so that the ROM of the hip joint is not limited in the latter half of the season.

There was no significant difference in the knee extension muscle strength. Since the rectus femoris, the primary knee extensor, has high durability against eccentric contraction [25], it is considered that the knee extension muscle strength did not fatigue easily. The hip extension muscle strength tended to decrease in the stride leg and significantly decreased in the push-off leg. This suggests that the season progression had a significant influence on the extensor muscles of bilateral hip joints. The H/Q ratio was clinically low in this study and suggested weakness of knee flexors. During throwing, the muscle activity of the biceps femoris, the primary knee flexor, is up to 142% maximum voluntary isometric contraction (MVIC) in the push-off leg and up to 125% MVIC in the stride leg [26]. The muscle strength of the hamstring was weaker than that of the quadriceps femoris, and any large requirements of muscle activities may have increased the risk of trauma, such as pulled hamstrings.

Finally, the number and speed of pitches are considered as risk factors for developing throwing disorders [27-29]. The shoulders and elbows of professional baseball pitchers, who are considered among the best, are always in danger. It has also been reported that the cumulative number of pitches affects the subsequent performance [30]. In this study, it was demonstrated that professional baseball pitchers had limitations in the ROM and developed muscle weakness in each of the upper limbs, trunk, and lower limbs as the season progressed. In order to prevent performance deterioration and development of throwing disorders due to the cumulative number of pitches, it is necessary to focus on parameters that showed significant differences as the season progressed and to appropriately train and condition these muscle groups.

The limitations of this study were that the sample size was small, the influence on the movement could not be verified, and the fatigue as the season progressed could not be quantified. If there were more participants, other significant parameters that affect throwing physical function may appear. Therefore, increasing the number of participants would be beneficial for addressing the other potential contributing factors to throwing disorders, such as pitching mechanics, workload management, and injury history. In addition, since only a static evaluation of the ROM and isometric muscle strength was carried out, we cannot speculate what kind of influence actually occurs on the throwing motion. Moreover, as the fatigue could not be quantified in this study, we could not establish whether this decrease in physical function was due to the accumulated fatigue or some other causes. Future studies with more participants that quantify fatigue and study its relationship with throwing movement are required.

## Conclusions

This study examined changes in the physical function of professional baseball pitchers during the season from the perspective of ROM and muscle strength. We found that the total shoulder arc ROM and shoulder abduction muscle strength of the dominant sides, trunk lateral flexion ROM and muscle strength, push-off leg hip extension and internal rotation ROM, and hip extension muscle strength were more susceptible to changes as the season progressed. In order to maintain performance and prevent the development of throwing disorders, it is necessary to focus on these movements during the season and to appropriately train and condition these muscle groups.
